# Digital Competencies and Training Approaches to Enhance the Capacity of Practitioners to Support the Digital Transformation of Public Health: Rapid Review of Current Recommendations

**DOI:** 10.2196/52798

**Published:** 2024-09-09

**Authors:** Swathi Ramachandran, Hsiu-Ju Chang, Catherine Worthington, Andre Kushniruk, Francisco Ibáñez-Carrasco, Hugh Davies, Geoffrey McKee, Adalsteinn Brown, Mark Gilbert, Ihoghosa Iyamu

**Affiliations:** 1 Clinical Prevention Services, British Columbia Centre for Disease Control, Vancouver, BC, Canada; 2 School of Public Health and Social Policy, University of Victoria, Victoria, BC, Canada; 3 School of Health Information Science, University of Victoria, Victoria, BC, Canada; 4 Dalla Lana School of Public Health, University of Toronto, Toronto, ON, Canada; 5 School of Population and Public Health, University of British Columbia, Vancouver, BC, Canada; 6 Population and Public Health, British Columbia Centre for Disease Control, Vancouver, BC, Canada

**Keywords:** digital public health, digital transformation, digital transformations, rapid review, rapid reviews, synthesis, review methods, review methodology, competencies, competency, training and practice recommendations, public health workforce, workforce, worker, workers, practitioner, practitioners, public health, digital health, training, continuing education, skills, skill, recommendation, recommendations, best practice, guideline, guidelines

## Abstract

**Background:**

The COVID-19 pandemic highlighted gaps in the public health workforce’s capacity to deploy digital technologies while upholding ethical, social justice, and health equity principles. Existing public health competency frameworks have not been updated to reflect the prominent role digital technologies play in contemporary public health, and public health training institutions are seeking to integrate digital technologies in their curricula.

**Objective:**

As a first step in a multiphase study exploring recommendations for updates to public health competency frameworks within the Canadian public health context, we conducted a rapid review of literature aiming to identify recommendations for digital competencies, training approaches, and inter- or transdisciplinary partnerships that can enhance public health practitioners’ capacity to support the digital transformation of public health.

**Methods:**

Following the World Health Organization’s (2017) guidelines for rapid reviews, a systematic search was conducted on Ovid MEDLINE, Ovid Embase, ERIC (Education Resources Information Center), and Web of Science for peer-reviewed articles. We also searched Google Scholar and various public health agency and public health association websites for gray literature using search terms related to public health, digital health, practice competencies, and training approaches. We included articles with explicit practice competencies and training recommendations related to digital technologies among public health practitioners published between January 2010 and December 2022. We excluded articles describing these concepts in passing or from a solely clinical perspective.

**Results:**

Our search returned 2023 titles and abstracts, of which only 12 studies met the inclusion criteria. We found recommendations for new competencies to enable public health practitioners to appropriately use digital technologies that cut across all existing categories of the core competencies for public health framework of the Public Health Agency of Canada. We also identified a new competency category related to data, data systems management, and governance. Training approaches identified include adapted degree-awarding programs like combined public health and informatics or data science degree programs and ongoing professional certifications with integration of practice-based learning in multi- and interdisciplinary training. Disciplines suggested as important to facilitate practice competency and training recommendations included public health, public health informatics, data, information and computer sciences, biostatistics, health communication, and business.

**Conclusions:**

Despite the growth of digital technologies in public health, recommendations about practice competencies and training approaches necessary to effectively support the digital transformation of public health remain limited in the literature. Where available, evidence suggests the workforce requires new competencies that cut across and extend existing public health competencies, including new competencies related to the use and protection of new digital data sources, alongside facilitating health communication and promotion functions using digital media. Recommendations also emphasize the need for training approaches that focus on interdisciplinarity through adapted degree-awarding public health training programs and ongoing professional development.

## Introduction

The context for public health training, practice, and research is rapidly evolving due to the widespread development and deployment of digital technologies in the field. The COVID-19 pandemic highlighted the importance of digital technologies in sustaining public health systems, especially in crises, and emphasized gaps in the workforce’s capacity to deploy digital technologies while upholding ethical, social justice, and health equity principles that are fundamental to public health [1-3]. To facilitate essential public health functions, digital technologies are increasingly being used, including artificial intelligence (AI), machine learning (ML), and digital dashboards, to enable real-time disease surveillance and predict population and public health outcomes [4-7]. Social media and other health apps and websites have enabled people-centered public health promotion and protection [4-7]. These use cases demonstrate potential benefits of digital technologies to ensure precise, proactive, and efficient public health services.

The public health workforce continues to encounter human resource capacity challenges regarding competencies to appropriately respond to the need for widespread integration of digital technologies within public health workstreams [8]. Growing public expectations for responsive and detailed public health information, influenced by experiences of digital transformation in sectors like finance, commerce, education, and health, generally further underscore the need for commensurate digital transformations of public health [3,9]. Digital transformation has been defined as a cultural shift involving the pervasive integration of digital technologies across organizational processes based on user needs [9]. The acceleration of digital transformation in society during the COVID-19 pandemic further accentuated the role of digital technologies as a “superdeterminant” of health and health disparities [10-13]. The public health workforce must develop new competencies to appropriately leverage digital technologies for public health functions, including monitoring and addressing the health implications arising from digital transformation in society [14]. While this transformation affects both public health and clinical medicine, the discourse around digital health is largely focused on clinical uses of digital technologies or virtual care (eg, telemedicine or remote patient monitoring), often leaving public health perspectives unattended. Despite an overlap between clinical medicine and public health, we need a distinct understanding of how public health—the organized effort of society to keep people healthy and prevent injury, illness, and premature death—might be impacted through digital technologies and what digital competencies are required to optimize public health outcomes [9,15].

In Canada, the public health core competency framework was last published in 2007 by the Public Health Agency of Canada (PHAC) and does not reflect current digital contexts [3,8,16]. It also does not adequately consider the prominent role digital technologies play in contemporary public health. In other jurisdictions, public health training institutions have considered integrating more advanced training on digital technologies within their public health curricula. In 2020, the World Health Organization and Association of Schools of Public Health in the European Regions updated their competency framework, emphasizing competencies for applying digital technologies for managing, analyzing, and storing health information and communicating health messages through modern (digital) media [17]. In the United States, the Council on Linkages between Academia and Public Health Practice updated its core competencies in 2021, maintaining competency statements like “applies public health informatics in using data, information and knowledge” in its data analytics and assessment domain, while adding similar statements related to social media use [18]. The existing frameworks do not describe disciplinary perspectives that can facilitate these competencies. Our previous work suggests interdisciplinary perspectives (including engineering, computer science, health information, and behavioral sciences) are crucial for these competencies [1,8], but the scope and characteristics of these interdisciplinary collaborations remain unclear.

This review aimed to contribute to the advancement of public health curricula, especially in Canada, by identifying practice competency and training recommendations to enhance public health practitioners’ capacity to support the digital transformation of public health. Here, we refer to practice competencies as the essential knowledge, skills, and attitudes necessary for the practice of public health that transcend disciplines and specific programs and form the baseline requirements to fulfill public health functions [16]. We use the term “public health practitioners” to refer to a broad range of professionals who work in various areas of public health and serve to improve health outcomes at a population level, as compared to the individual level (these professionals comprise the public health workforce). They include, but are not limited to, public health nurses and physicians, epidemiologists, health promotion specialists, and public health managers [19]. We also aimed to identify disciplinary perspectives relevant to implementing practice competency and training recommendations and the scope of interdisciplinary partnerships that may be required.

## Methods

### Overview

We conducted our rapid review following the Cochrane rapid review recommendations [20] and World Health Organization guidelines [21]. Our rapid review protocol was registered on PROSPERO (CRD42022384474) and published on the Open Science Framework [22]. This review is the first phase of a multiphase study aiming to contribute recommendations for digital public health workforce competencies in Canada [23]. Given the exploratory nature of our research, our reporting aligned best with the Preferred Reporting Items for Systematic Reviews and Meta-Analyses Extension for Scoping Reviews (PRISMA-ScR) (Multimedia Appendix 1 [24]). Our focused research questions were as follows: (1) What public health practice competencies are recommended to facilitate the integration of digital technologies in public health functions? (2) What training approaches may best facilitate the learning and teaching of identified practice competency recommendations? and (3) What disciplines/disciplinary perspectives should be considered to facilitate identified practice competencies and training recommendations?

### Data Sources

We searched Ovid MEDLINE, Ovid Embase, Web of Science, and ERIC (Education Resources Information Center) for peer-reviewed articles exploring recommendations for practice competencies and training related to digital technologies for public health. In consultation with a University of British Columbia librarian, we created a search strategy (Table 1) with terms related to public health (defined as the organized effort of society to keep people healthy and prevent injury, illness, and premature death through a combination of programs, services and policies that protect and promote the health of all) [15]; digital health (the field of knowledge and practice associated with the use of digital technologies to improve health) [25]; and practice competencies and training (essential knowledge, skills, and attitudes necessary for the practice of public health that transcend disciplines and specific programs and form the baseline requirements to fulfill public health functions) [16]. We also searched various public health agency websites (Multimedia Appendix 2) and preprint servers such as Europe PubMed Central (PMC), which indexes preprints, including those from arXiv.org, for gray literature. We conducted a supplementary search on Google Scholar and Google using a simplified version of our original search and explored the first 10 pages of returned results, as is commonly done in similar reviews [26,27]. Finally, we used CitationChaser to perform automated forward and backward citation searches for articles included in our study [28]. Our search was limited to articles published in English between January 2010 and December 13, 2022 (the date of our search). All relevant search returns were exported to Covidence (Veritas Health Innovation) for citation management and review.

**Table 1.** Search terms for rapid review.

Public health^a^	Public health, epidemiolog*, surveillance, “health promotion,” “health protection,” “health prevention,” “health policy,” “community health,” “health evaluation,” “emergency preparedness,” “health equity”
Digital health^b^	“Digital health,” “digital public health,” “digital tech,*” “health informatics,” “biomedical informatics,” “medical informatics,” mhealth, ehealth, e-health, “artificial intelligence,” “machine learning,” “big data,” “social media,” digitalization, “digital transformation”
Training and practice competencies	Learn*, train*, educat*, competenc*, skill, course, qualification, faculty, aptitude, capacity, curricul^*^

a Terms related to public health were derived from the domains of public health described by the Canadian Public Health Association. A limited number of terms were used to ensure a manageable number of search returns.

b Terms related to digital health were selected based on the European Public Health Association’s conceptual framework for digital public health, which informed our digital public health framework. The technologies most applied in public health domains were selected alongside terms describing digital technologies more generally.

### Screening and Selection Procedure

Our inclusion and exclusion criteria were informed by our adaptation of the Joanna Briggs Institute PCC (population, concept, and context) research question framework (Textbox 1) [29]. We included publications describing practice competencies and training recommendations related to the use of digital technologies for population and public health among public health students, public health informaticians and analysts, public health physicians, practitioners, researchers, and decision-makers [30]. We included reviews, commentaries, position statements, policy guidelines, and primary research studies exploring our concept of interest. We excluded publications with a solely clinical perspective.

Textbox 1. **Inclusion and exclusion criteria for selected papers exploring digital competencies for public health, including population, concept, context, time frame, study design, and language. These domains were based on the adapted Joanna Briggs Institute PCC (population, concept, and context) research question framework. Additions include time frame, study design, and language. The public health workforce was defined as all workers engaged in public health activities who identify public health as being their primary role. This includes public health consultants and specialists, directors of public health, public health nurses, health visitors, school nurses, public health practitioners, environmental health professionals, public health academics, scientists, and intelligence and knowledge professionals.**

**Inclusion criteria**

- Population: public health students, public health informaticians and analysts, practitioners (including public health physicians), public health researchers, decision- and policy makers
- Concept: articles describing training and practice competencies related to digital technologies; articles describing disciplines relevant in facilitating training and practice competencies related to digital technologies; articles describing approaches and outcomes of training related to digital technologies
- Context (public health defined by the domains proposed by the Canadian Public Health Association): population and public health
- Time frame: January 2010 December 13, 2022
- Study design: reviews, perspectives, or commentaries; primary research studies, including quantitative (eg, cross-sectional studies) and qualitative studies; position statements by relevant stakeholders; public health agency policy and guideline documents and reports
- Language: published in English

**Exclusion criteria**

- Population: exclusive focus on clinicians, programmers, informaticians, or other allied health system practitioners
- Concept: articles describing training in passing or as part of a broader discussion of other health issues
- Context: clinical or solely clinical contexts
- Time frame: published before 2010
- Study design: articles without full texts (>500 words) available
- Language: published in any language other than English

**Figure 1.**
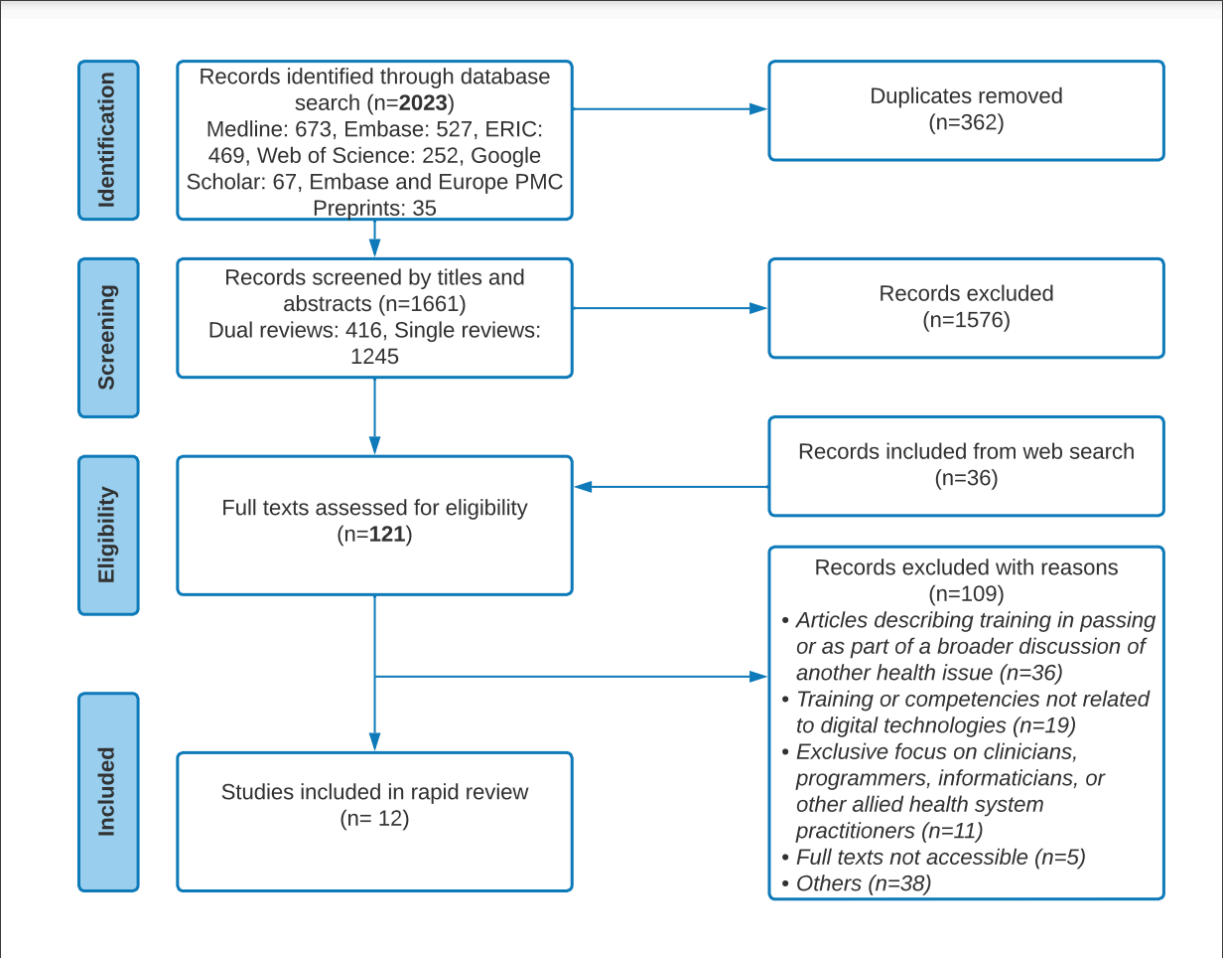
Flow diagram of the search and study selection process following the PRISMA (Preferred Reporting Items for Systematic Reviews and Meta-Analysis) guidelines for scoping reviews. ERIC: Education Resources Information Center; PMC: PubMed Central.

We conducted our screening and selection in 2 phases after automatic deduplication on Covidence. For feasibility and in line with Cochrane rapid review recommendations, 2 reviewers (SR and II) independently screened 25% of the titles and abstracts using the inclusion and exclusion criteria, regularly meeting to discuss discrepancies and achieve consensus. The remaining titles and abstracts were screened by 1 reviewer (SR). In the second phase, both reviewers (SR and II) assessed the eligibility of all included full texts, meeting to discuss discrepancies where appropriate. A summary of the selection process is described in Figure 1.

### Empirical Critical Appraisal of Included Studies

Considering the mixed nature of the included publications, we only conducted a risk of bias assessment of the primary research studies included in this study, using the Mixed Methods Appraisal Tool (version 2018) (Multimedia Appendix 3 [31-43]).

### Data Extraction and Analyses

We extracted data from the included publications using pretested data extraction forms in Covidence (Multimedia Appendix 4) [15]. These forms were tested using the first 2 included publications, with discussions between reviewers to clarify discrepancies in interpretation. We extracted bibliographic information including authors’ names, year of publication, title, journal, country and continent of first author, publication type, and study aims or objectives (where applicable). We also extracted competency recommendations and mapped them onto the existing competency categories of the 2007 PHAC framework [32-43] for core competencies for public health and created new categories for recommendations not fitting into existing categories [16]. For competency recommendations applicable to more than 1 category, we mapped the recommendations into multiple categories. We further categorized the competencies as those specific to digital technologies, those not specific but relevant to digital technologies, and basic literacy competencies specific to digital technologies. Finally, we extracted descriptions and recommendations about training approaches, disciplines involved in facilitating training, and public health domains where competency recommendations were most applicable [15]. All these were summarized using a narrative synthesis.

## Results

### Characteristics of Included Publications

Table 2 describes the 12 publications included in this review, all by authors in North America [32-43]; 11 of 12 were written by first authors in the United States [32,34-43], 5 of 12 were published between 2010 and 2015 [34,37,38,40,43] and 6 of 12 were commentaries or perspectives [33,35,36,40-42]. Of these, the 4 studies included in the quality appraisal showed variable quality, with 3 being observational studies (Multimedia Appendix 3).

**Table 2.** Characteristics of publications exploring digital competencies for public health as included in the rapid review.

Characteristics		Publications (n=12), n (%)
**Country of first author**		
	Canada	1 (8)
	United States	11 (92)
**Year of publication**		
	2010‐2015	5 (42)
	2016‐2020	4 (33)
	2021‐2022	3 (25)
**Publication type**		
	Commentaries/perspectives	6 (50)
	Cross-sectional studies	3 (25)
	Environmental scans	2 (17)
	Literature review and key informant interviews	1 (8)
**Public health domains referenced** ^**a**^		
	Research	10 (83)
	Epidemiology and surveillance	9 (75)
	Risk assessment	3 (25)
	Health promotion	3 (25)
	Evaluation	2 (17)
	Community consultation	2 (17)
	Health policy	1 (8)

a Terms derived from the domains of public health described by the Canadian Public Health Association.

### Domains of Public Health Relevant to Digital Technologies

Practice competencies were most described in relation to public health research (n=10 articles) [32-40,42,43], epidemiology and surveillance (n=9 articles) [32-36,38,39,42,43], risk assessment (n=3 articles) [32,33,38], and health promotion (n=3 articles) [38,41,43].

### Practice Competency Recommendations Related to Digital Technologies

We identified competency recommendations regarding the implementation of digital technologies across all 7 competency categories in the PHAC framework (Table 3). We also identified an additional category of competencies for managing digital data, data systems management, and governance. Here, we highlight *competency statements that are not specific to, but relevant to, digital technologies* in public health, including using multiple data sources (including digital data) to answer public health questions (public health sciences), understanding methods for data linkage (assessment and analyses), ensuring inter- and transdisciplinary methods for designing and implementing studies and research (partnerships, collaboration, and advocacy), and developing an entrepreneurial orientation to innovation and risk-taking to address public health concerns (leadership) [16]. Others include defining audiences for developing and disseminating tailored public health information based on trusted formats and information sources (communication) and developing tools to protect the privacy of individuals and communities involved in public health programs (data and data systems management and governance). These competency statements serve as an overview of what was identified from the literature and can serve as a guide in determining important considerations in updating public health curricula.

**Table 3.** Competency recommendations identified in included publications in relation to digital technologies, mapped to the core competencies for public health in Canada.

Competency category	Competency statements	A public health practitioner can...
Public health sciences: this includes key knowledge and critical thinking skills related to the public health sciences	Competencies specific to digital technologies	Develop and ethically apply research methods using data science, statistical genetics, and “omics” technologies (eg, exposomes); computational biology; and epidemic and infectious disease modeling to address public health problems [32-35,37,40]. Conduct education and training in public health informatics [38]. Identify opportunities to incorporate emerging omics technologies into epidemiological research [27,34].^a^
	Competencies not specific to, but relevant to digital technologies	Use different types of data (including digital data sources) to answer public health questions [40,42]. Aid public health organizations to design and test new data systems [42].
Assessment and analysis: this category relates to the core competencies needed to collect, assess, analyze, and apply information	Competencies specific to digital technologies	Use, protect, and interpret complex, linked large data sets at various levels of data organization, including administrative, clinical, biological, environmental, population, and social or societal levels from multiple sources within and outside the health system [32-34,37,38,42,43]. Design, analyze, and report health science data by blending traditional and modern analytic and computational techniques (eg, biostatistics, informatics, computer-based programming, and software, as appropriate [Tableau, python, MS-SQL, R]) [32-34,39,42]. Identify needs, challenges, principles, and key details for data sharing and large-scale data management [34,35,38,42]. Use and interpret findings from data exploration tools and analytic approaches used in computer sciences, such as natural language processing [34,38]. Use information technologies and communication tools necessary to support epidemiologic investigations and surveillance (eg, smartphone surveys, GPS tracking) [34,35]. Understand how omics tools can be integrated into an ecological model of health including social and environmental determinants [35,43].^a^ Identify the role of emerging omics technologies applicable to epidemiologic research and incorporate them into epidemiologic research [35,43].
	Competencies not specific to, but relevant to digital technologies	Know and understand methodological and statistical problems inherent in secondary and big data analyses [34]. Understand methods for linking data sources to create information [34,35,37].
Policy and program planning, implementation, and evaluation: competencies essential in effectively choosing options and planning, implementing, and evaluating policies and programs in public health	Competencies specific to digital technologies	Design, implement, and evaluate population-based projects, programs, or interventions that use social media for communication, public health education, and promotion [36]. Support use of informatics to promote disease prevention at the clinical health, environmental, and personal health interface [37]. Design, develop, and implement user-centered population health information systems [38,39]. Support information system development, procurement, and implementation that meets public health program needs [38,39,42]. Establish frameworks for evaluating information systems and application implementations and make recommendations to improve user satisfaction and outcomes [38,39,42]. Evaluate communication, that is, assess the reach and dose of communication to various public health audiences using tools including website and social media analytics, data mining software, focus groups, and in-depth interviews [34,35,41].
	Competencies not specific to, but relevant to digital technologies	Develop tools that protect the privacy of individuals and communities involved in health programs, policies, and research [35].
Partnerships, collaboration, and advocacy: this category captures the competencies required to influence and work with others pursuing the common goal to improve the health and well-being of the public	Competencies specific to digital technologies	Use new media, such as social media, to conduct advocacy [41]. Manage IT operations related to projects or programs and those managed by external organizations [38].
	Competencies not specific to, but relevant to digital technologies	Assess stakeholder data, information, and knowledge needs [39,42]. Develop team approaches bringing together diverse disciplines and organizations to develop new and creative ways of designing and implementing studies and addressing public health concerns [34,38,41].
Diversity and inclusion: sociocultural competencies required to interact effectively with diverse individuals, groups, and communities	Competencies not specific to, but relevant to digital technologies	Develop team approaches that bring together diverse disciplines and organizations to develop new and creative ways of designing and implementing studies and addressing public health concerns [34,38,41].
Communication: addresses dimensions of communication including internal and external exchanges; written, verbal, nonverbal, and listening skills; computer literacy; providing appropriate information to different audiences; working with the media; and social marketing techniques.	Basic literacy competencies specific to digital technologies	Effectively communicate with teams through email, word processing, spreadsheet, and presentation software [32,37].
	Competencies specific to digital technologies	Use mass media, electronic technology, and communication methods (eg, social media) for public health communication, health education, and promotion [34,36,41]. Evaluate communication, that is, assess the reach and dose of communication to various public health audiences using tools including website and social media analytics, data mining software, focus groups, and in-depth interviews [34,35,41].^a^
	Competencies not specific to, but relevant to digital technologies	Use evidence-based communication program models to disseminate research and evaluation outcomes [34,35]. Define target audiences and develop appropriate messaging to reach people where they are, considering the people, places, and media they interact with and their trusted information sources and formats [34-36,41]. Tailor surveillance information content and periodicity of dissemination for specific audiences and their uses [34,35].
Leadership: focuses on leadership competencies that build capacity, improve performance, and enhance the quality of the working environment	Competencies specific to digital technologies	Support development of strategic directions for public health informatics within the enterprise [38]. Address IT development using principles of systems thinking and engineering [36-39]. Communicate public health informatics needs and proposed solutions to leaders in public health and elected officials effectively [37,42]. Contribute to considerations on the best IT systems for the public health enterprise to use [37]. Manage and direct health informatics planning for projects related to public health and information technology [38,42].
	Competencies not specific to, but relevant to digital technologies	Show entrepreneurial orientation through proactiveness, innovativeness, and risk-taking to advance public health, address newly emerging public health issues, and better understand risks when aligning community health partners to address health inequities [36].
Data and data systems management and governance: competencies relevant in the design and management of new digital data streams and ensuring appropriate, ethical use of the data for public health goals while protecting health users’ privacy and confidentiality	Competencies specific to digital technologies	Apply procedures (policies) and technical means (security) to ensure the integrity and protection of confidential information in electronic files and computer systems while maximizing the benefits to public health [34,35,38,39,42]. Contribute to developing public health information systems that are interoperable with other relevant information systems [38,42]. Manage data and information in compliance with policies, protocols, and informatics standards [38,42]. Aid public health organizations in thinking through and designing testing strategies for new data systems [42]. Describe systems analysis methods, requirements, use cases, data flow diagrams, business process modeling, data modeling, relational data theory, normalization, entity relationship diagrams, SQL, and data warehouses [42]. Describe conceptualizations of data, medical vocabulary nomenclature, terminologies, coding and classification, standards, ethics, privacy, security, health systems, computer technologies, health information exchange, data quality, and information architectures [42]. Create systems for health information exchange between health organizations and databases [42].
	Competencies not specific to, but relevant to digital technologies	Develop tools that protect the privacy of individuals and communities involved in health programs, policies, and research [34,35,39]. Document business rules using algorithms and pseudocode [42]. Assess and manage risks associated with using and sharing data and information, data security, and intellectual property [42].

a Duplicated across various applicable competency categories.

Regarding *competencies specific to digital technologies* in public health, the most cited competency statements related to developing and ethically applying modern research methods requiring big data (eg, omics and computational biology in public health sciences) [35] and tools (eg, computer programming and geographic information systems) [32]; using, protecting, and interpreting large linked data sets from multiple sources using a blend of traditional and modern analytic techniques (assessment and analyses) [34,35,37]; communicating with the public using tools like social media for public health (communications) [36,41]; and establishing frameworks for evaluating health information systems and communication efforts using tools like social media and web analytics (communication, policy and program planning, implementation, and evaluation) [41]. Additional competencies in this category include supporting the development of IT systems using systems thinking (leadership) [37,39], applying procedures and technical means to ensure the integrity and protection of confidential information in electronic files and computer systems, and developing interoperable data systems (data and data systems management and governance) [34,35,38].

One recommendation was identified for *basic literacy competencies specific to digital technologies* regarding effectively communicating with teams through email, word processing, spreadsheets, and presentation software (communication) [32].

### Training Approaches

The 2 main categories of training approaches identified included recommendations for adapted degree-awarding programs and ongoing training or professional development. Most of the recommendations concerned adapting degree-awarding public health programs. These included expanded public health core training on digital technologies as introductory training with elective specialization [33-36,38,40,43], practice-informed training embedded in real-world contexts and organizations [33,41,42], specialized master’s- and doctoral-level training in specific digital technology–related fields like data science and population and public health informatics [33,34,39], integrated master’s in public health and public health informatics training programs, one-on-one mentorship programs in practice and research settings [34], and engaging interdisciplinary teams to design public health training for digital technologies [36]. The second category of training recommendations related to ongoing training and professional development in the context of continuing education [32,34,35,38,39]. These recommendations mainly included competency-based learning on digital technologies through short courses, workshops, and certification programs in partnerships with academic institutions [32,34,35] and online training targeting specific global workforce deficiencies [38,39].

### Disciplines Relevant in Implementing Digital Competencies and Training Programs for the Digital Transformation of Public Health

Disciplines identified as relevant to digital public health training include public health [32-36,41,43], public health informatics [32,34,35,37-40,42], computer science [33-35,40,42], data science [33-35], mathematics [33,40], statistics/biostatistics [33,40,43], health communication [34-36,43], sociology/social sciences [34,35,40], business [36], economics [36], management sciences [36,40,42], and information sciences [36,42,43].

## Discussion

### Principal Findings

In this rapid review, we identified 12 publications describing 45 unique recommendations for digital practice competencies across 8 competency categories described as essential to support the public health workforce in the digital transformation of public health, especially from a Canadian perspective. Recommendations cut across all competency categories of the 2007 public health core competency framework in Canada. These recommendations included basic digital literacy competencies (general digital skills required for carrying out public health operations), competencies specific to digital technologies (ie, competencies relevant to specific public health functions that leverage digital technologies), and competencies not specific to, but relevant to digital technologies (ie, broad competencies not specific to, but accentuated by using digital technologies in public health). We also identified recommendations related to a new competency category that we called “data and data systems management and governance” related to the management and use of new digital data relevant for public health functions.

Interestingly, our study identified gaps in the current literature. Despite demonstrable awareness of digital competency gaps for public health, substantive competency recommendations were generally lacking, as evident with only 12 articles included in this study. The digital competency discourse was also dominated by “public health informatics” and “data science,” as 8 of the 12 articles were focused on “public health informatics.” While partly attributable to the subsuming of digital public health into the broader discourse on digital health, it demonstrates the need for research in this space. Recent “digital health” reviews have identified diverse competencies like understanding concepts for AI and ML more specifically in health, modern data infrastructure, pros and cons of telehealth, applications of wearable sensors, and skills for managing health services in the context of digital transformation [44,45]. Researchers suggest that similar competencies focused on “digital public health” are necessary to ensure that the digital transformation of public health is not overlooked, as it is a distinct but related field to digital health [14,46].

This study also identified training recommendations, including adapting degree-awarding programs and ongoing professional development to integrate interdisciplinary approaches to ensure broad competencies for digital technologies with options for specialized competency-based training where necessary. These findings parallel those of a scoping review of digital health competencies for medical school education that suggested recognition of interdisciplinary collaboration in digital health is an important attitude to be considered in contemporary training [36,44]. As identified in our study, such disciplines could include public health (including epidemiology and health promotion), health informatics (public health informatics), information sciences, computer science, biostatistics, mathematics, management sciences, health communication, sociology, and other similar disciplines. Few jurisdictions have invested in exploring these interdisciplinary perspectives. The United Kingdom’s National Health Service initiated the Graduate Management Training Scheme for health systems leaders with a health informatics specialization [45]. The Australasian Institute of Digital Health notes similar interdisciplinary capacity-building efforts are needed to prepare the health workforce to address the impacts of digital technologies [45,47]. While these efforts have been within the broader field of digital health, similar interdisciplinary training efforts are required to translate the leadership competency recommendations identified in this study.

To the best of our knowledge, this rapid review represents the first review of evidence on digital competency and training recommendations specifically for public health. While we have used the framework of the core competencies as developed in Canada by the PHAC to situate the training recommendations, these competencies and training recommendations have implications not just in Canada, but across other jurisdictions. First, there is a critical evidence gap that must be filled concerning digital competencies for public health even within the broader digital health discourse [48,49]. Our findings highlight current recommendations while demonstrating the relative inattention to the unique competencies required for the digital transformation of public health practice. Second, this review highlights the need for generalized orientation of public health practitioners to digital technologies, while creating opportunities for specialized digital competencies, especially through practice-based learning. Such approaches will enhance deeper understanding of the challenges these technologies pose, while contextualizing jurisdictional policies relevant to the ethical and just use of these technologies, especially the use of large digital data sets pulling health information from various sources; designing, implementing, and evaluating people-centered digital innovations in public health; using social media for targeted communication; providing leadership in managing digital technologies for public health; and communicating the need for these tools with the public and relevant partners [6]. Third, this review highlights the need to deeply integrate interdisciplinarity in the public health curriculum. This imperative will not only allow public health practitioners to adequately use digital technologies for their functions but prepare them to respond to digital technologies as a superdeterminant of health [9]. For example, the World Health Organization has suggested competency frameworks for managing infodemics—the spread of misinformation and disinformation, which is amplified by widespread access to unverified information online [50]. There are also advantages of strengthening interdisciplinary digital public health curricula, as these could have multiplier effects in building public capacity given the role public health practitioners play in educating the public on health threats—including mis- and disinformation.

Fourth, this review also identified digital competencies and gaps requiring additional research and training approaches that leverage interdisciplinarity. We described competencies not specific to, but related to, digital technologies in public health that mostly reflect attitudinal changes required of the workforce to effectively leverage digital technologies and respond to future technological changes. For example, there are recommendations for additional social, emotional, and cognitive competencies, including curiosity, innovation, willingness to make mistakes but learn from them, and interdisciplinarity—a paradigm well aligned with human-centered approaches to developing digital technologies more broadly but currently at odds with public health’s risk-averse stance [51,52]. Finally, beyond research on new digital competencies from a public health perspective, additional research is needed to effectively integrate identified recommendations into current competency frameworks. Such research must consider components of generalized curricular updates to help practitioners understand the digital landscape while creating space for specialized training programs for specific public health roles [44]. Most existing competency frameworks promote a tiered approach, where frontline workers with digital technologies have more specific training compared with those in management positions, who require a basic understanding of how the digital technologies work [18]. Given escalating demands for new competencies across various domains of public health, the challenge will be to holistically integrate these digital practices in existing curricula as opposed to simply attaching new courses. Specific attention will be required to address the newly identified competency category to ensure that principles of health equity and ethical propriety are upheld throughout the competencies. While our research aims to contribute to closing this knowledge gap, other similar research is ongoing (at the time of writing) in other jurisdictions, including updates to health management competencies for digital transformation in Australasia. However, a perspective lacking in these efforts involves mechanisms for ongoing and nimbler competency and curricular evaluations and updates, given the speed of evolution of digital technologies and the scope of their impacts on public health. Such mechanisms could involve a standing competency development committee with mandates for regular (perhaps yearly) minor updates, with larger updates requiring broad consultations considered on a longer timeframe. As a next step in our multiphase research, we are validating identified competency recommendations with public health practitioners through focus groups to ensure they are well adapted to the Canadian context while understanding the implications of such nimble update systems for their practices [14].

### Strengths and Limitations

This report represents a first attempt at synthesizing competency and training recommendations for improving the public health workforce’s capacity to effectively use digital technologies. Our exploration of competencies related to digital technologies specifically within public health is bolstered by our approach to identifying training recommendations and disciplinary approaches to achieve identified competencies. The rapid review methodology was used for pragmatic reasons because we intended to develop recommendations to inform focus groups in a larger project in Canada to ensure our academic and public health practice partners and knowledge users could consider updates to the curriculum. The Cochrane rapid review methodology recommends that 2 reviewers should perform dual screening of at least 20% of abstracts during title and abstract screening and perform a single review of full texts [20]. To ensure rigor, we dual-screened 25% of titles and abstracts and dual-screened all full texts (above recommended levels). We also searched multiple databases in line with recommendations, ensuring comprehensiveness of the review [53]. However, our rapid review is unlikely to have exhaustively characterized the research area, given our need for expedient delivery of evidence. Given the search strategy and inclusion criteria specifically identified publications discussing digital competencies and training approaches related to the role of digital technologies in executing a broad range of public health functions, we may have inadvertently excluded relevant competency recommendations made solely in the clinical context or within the broader context of digital health. Additionally, articles describing training in passing were not included, and this may have limited the breadth of digital competency recommendations identified. To ensure feasibility, we only included publications in English, and the review could have missed important non-English resources. Our broad approach and definitions of terms like “digital technologies in public health” may have also missed the depth of discussions within specific domains of public health. Finally, the timing of our search may have missed additional perspectives on digital competencies, given known publication lags and a refocus on better public health preparedness toward the end of the COVID-19 pandemic [54]. This is plausible given our findings of heightened awareness of digital competency gaps and extended periods required to update competency recommendations. Our recommendations for nimbler mechanisms for developing and updating competencies may address this issue.

### Conclusion

To adequately support the digital transformation of public health, practitioners require new digital competencies cutting across and extending existing public health competency frameworks, particularly in Canada. However, the literature is currently sparse regarding what these competencies are and what training approaches might facilitate the competencies. Therefore, research in this area is crucially needed alongside developing mechanisms for competency updates that attempt to keep pace with the development of digital technologies in public health. Where available, evidence suggests the workforce requires new competencies that cut across and extend existing categories of public health competency frameworks, including new competencies related to the use and protection of new digital data sources, alongside facilitating health communication and promotion functions using digital media. Recommendations also emphasize the need for training approaches that focus on interdisciplinarity and innovation through adapted degree-awarding public health training programs and ongoing professional development. As a next step, we are validating identified competency recommendations with public health practitioners to ensure they are well adapted to the Canadian context through focus group discussions with the Canadian public health workforce.

## Supplementary material

10.2196/52798 Multimedia Appendix 1 PRISMA-ScR (Preferred Reporting Items for Systematic Reviews and Meta-Analyses extension for Scoping Reviews) checklist.

10.2196/52798 Multimedia Appendix 2 Detailed search strategy for the rapid review.

10.2196/52798 Multimedia Appendix 3 Critical appraisal of included studies.

10.2196/52798 Multimedia Appendix 4 Data extraction form.

## Abbreviations

AI: artificial intelligence
ERIC: Education Resources Information Center
ML: machine learning
PCC: population, concept, context
PHAC: Public Health Agency of Canada
PMC: PubMed Central
PRISMA-ScR: Preferred Reporting Items for Systematic Reviews and Meta-Analyses Extension for Scoping Reviews
