# Supplementary material for: Digital Competencies and Training Approaches to Enhance the Capacity of Practitioners to Support the Digital Transformation of Public Health: Rapid Review of Current Recommendations
Source: JMIR Public Health Surveill. 2024 Sep 9;10:e52798. doi: 10.2196/52798 (PMC11403915; doi:10.2196/52798)
Supplement: Multimedia Appendix 2 [file publichealth-v10-e52798-s002.docx]

APPENDIX –

Detailed Search Strategy for Rapid review

Number of Peer-reviewed Documents Identified from Systematic Search

| Medline | 673 |
| --- | --- |
| Embase | 527 |
| ERIC | 469 |
| Web of Science | 252 |
| Google scholar | 59 |
| Google | 8 |
| Embase preprints | 35 |
| Europe PMC preprints | 28 |
| Duplicates | 362 |
| Total number of titles and abstracts to screen (in Covidence) | 1661 |

Detailed database search and results from OVID Medline

| 1 | Public Health/ | 93140 |  |
| --- | --- | --- | --- |
|  |  |  |  |
| 2 | public health.ti,kf. | 117457 |  |
|  |  |  |  |
| 3 | public health.ab. /freq=2 | 43221 |  |
|  |  |  |  |
| 4 | surveillance.ab. /freq=2 | 48321 |  |
|  |  |  |  |
| 5 | epidemiolog*.ab. /freq=2 | 60729 |  |
|  |  |  |  |
| 6 | Health Promotion.ab. /freq=2 | 7523 |  |
|  |  |  |  |
| 7 | health protection.mp. | 4205 |  |
|  |  |  |  |
| 8 | health prevention.mp. | 1804 |  |
|  |  |  |  |
| 9 | health policy.mp. | 94063 |  |
|  |  |  |  |
| 10 | community health.mp. | 89745 |  |
|  |  |  |  |
| 11 | health evaluation.mp. | 7718 |  |
|  |  |  |  |
| 12 | emergency preparedness.mp. | 2528 |  |
|  |  |  |  |
| 13 | health equity.mp. | 8753 |  |
|  |  |  |  |
| 14 | digital health.mp. | 6295 |  |
|  |  |  |  |
| 15 | digital public health.mp. | 61 |  |
|  |  |  |  |
| 16 | digital tech*.mp. | 5234 |  |
|  |  |  |  |
| 17 | health informatics.mp. | 4593 |  |
|  |  |  |  |
| 18 | biomedical informatics.mp. | 914 |  |
|  |  |  |  |
| 19 | public health informatics.mp. | 1479 |  |
|  |  |  |  |
| 20 | medical informatics.mp. | 18158 |  |
|  |  |  |  |
| 21 | mhealth.mp. | 8414 |  |
|  |  |  |  |
| 22 | ehealth.mp. | 6439 |  |
|  |  |  |  |
| 23 | e-health.mp. | 3927 |  |
|  |  |  |  |
| 24 | artificial intelligence.mp. | 50859 |  |
|  |  |  |  |
| 25 | machine learning.mp. | 82190 |  |
|  |  |  |  |
| 26 | big data.mp. | 12967 |  |
|  |  |  |  |
| 27 | social media.mp. | 30500 |  |
|  |  |  |  |
| 28 | digitalization.mp. | 2193 |  |
|  |  |  |  |
| 29 | digital transformation.mp. | 789 |  |
|  |  |  |  |
| 30 | train*.ab. /freq=2 | 268326 |  |
|  |  |  |  |
| 31 | education*.ab. /freq=2 | 194781 |  |
|  |  |  |  |
| 32 | public health professional.mp. | 982 |  |
|  |  |  |  |
| 33 | professional competenc*.mp. | 26868 |  |
|  |  |  |  |
| 34 | competenc*.ab. /freq=2 | 31933 |  |
|  |  |  |  |
| 35 | skill.mp. | 50904 |  |
|  |  |  |  |
| 36 | course.ab. /freq=2 | 90471 |  |
|  |  |  |  |
| 37 | qualification.mp. | 9904 |  |
|  |  |  |  |
| 38 | faculty.mp. | 80043 |  |
|  |  |  |  |
| 39 | aptitude.mp. | 8624 |  |
|  |  |  |  |
| 40 | curricul*.mp. | 119622 |  |
|  |  |  |  |
| 41 | 1 or 2 or 3 or 4 or 5 or 6 or 7 or 8 or 9 or 10 or 11 or 12 or 13 or 32 | 476550 |  |
|  |  |  |  |
| 42 | 14 or 15 or 16 or 17 or 18 or 19 or 20 or 21 or 22 or 23 or 24 or 25 or 26 or 27 or 28 or 29 | 203633 |  |
|  |  |  |  |
| 43 | 30 or 31 or 32 or 33 or 34 or 35 or 36 or 37 or 38 or 39 or 40 | 733437 |  |
|  |  |  |  |
| 44 | 41 and 42 and 43 | 761 |  |
|  |  |  |  |
| 45 | limit 41 to (English language and yr="2010 - current") | 673 |  |

Detailed database search and results from OVID EMBASE

| 1 | Public Health/ | 218680 |  |
| --- | --- | --- | --- |
|  |  |  |  |
| 2 | public health.ti,kf. | 88341 |  |
|  |  |  |  |
| 3 | public health.ab. /freq=2 | 49853 |  |
|  |  |  |  |
| 4 | surveillance.ab. /freq=2 | 68979 |  |
|  |  |  |  |
| 5 | epidemiolog*.ab. /freq=2 | 75978 |  |
|  |  |  |  |
| 6 | Health Promotion.ab. /freq=2 | 8668 |  |
|  |  |  |  |
| 7 | health protection.mp. | 5012 |  |
|  |  |  |  |
| 8 | health prevention.mp. | 2214 |  |
|  |  |  |  |
| 9 | health policy.mp. | 38069 |  |
|  |  |  |  |
| 10 | community health.mp. | 59068 |  |
|  |  |  |  |
| 11 | health evaluation.mp. | 9886 |  |
|  |  |  |  |
| 12 | emergency preparedness.mp. | 2993 |  |
|  |  |  |  |
| 13 | health equity.mp. | 11771 |  |
|  |  |  |  |
| 14 | public health professional.mp. | 183 |  |
|  |  |  |  |
| 15 | digital health.mp. | 6239 |  |
|  |  |  |  |
| 16 | digital public health.mp. | 58 |  |
|  |  |  |  |
| 17 | digital tech*.mp. | 7008 |  |
|  |  |  |  |
| 18 | health informatics.mp. | 3785 |  |
|  |  |  |  |
| 19 | biomedical informatics.mp. | 1042 |  |
|  |  |  |  |
| 20 | public health informatics.mp. | 339 |  |
|  |  |  |  |
| 21 | medical informatics.mp. | 25214 |  |
|  |  |  |  |
| 22 | mhealth.mp. | 7638 |  |
|  |  |  |  |
| 23 | ehealth.mp. | 6496 |  |
|  |  |  |  |
| 24 | e-health.mp. | 5241 |  |
|  |  |  |  |
| 25 | artificial intelligence.mp. | 56387 |  |
|  |  |  |  |
| 26 | machine learning.mp. | 110600 |  |
|  |  |  |  |
| 27 | big data.mp. | 15153 |  |
|  |  |  |  |
| 28 | social media.mp. | 47892 |  |
|  |  |  |  |
| 29 | digitalization.mp. | 3229 |  |
|  |  |  |  |
| 30 | digital transformation.mp. | 760 |  |
|  |  |  |  |
| 31 | train*.ab. /freq=2 | 370477 |  |
|  |  |  |  |
| 32 | education*.ab. /freq=2 | 270899 |  |
|  |  |  |  |
| 33 | professional competenc*.mp. | 35783 |  |
|  |  |  |  |
| 34 | competenc*.ab. /freq=2 | 39830 |  |
|  |  |  |  |
| 35 | skill.mp. | 165654 |  |
|  |  |  |  |
| 36 | course.ab. /freq=2 | 139978 |  |
|  |  |  |  |
| 37 | qualification.mp. | 15175 |  |
|  |  |  |  |
| 38 | faculty.mp. | 80466 |  |
|  |  |  |  |
| 39 | aptitude.mp. | 9739 |  |
|  |  |  |  |
| 40 | curricul*.mp. | 137499 |  |
|  |  |  |  |
| 41 | 1 or 2 or 3 or 4 or 5 or 6 or 7 or 8 or 9 or 10 or 11 or 12 or 13 or 14 | 524770 |  |
|  |  |  |  |
| 42 | 15 or 16 or 17 or 18 or 19 or 20 or 21 or 22 or 23 or 24 or 25 or 26 or 27 or 28 or 29 or 30 | 260737 |  |
|  |  |  |  |
| 43 | 31 or 32 or 33 or 34 or 35 or 36 or 37 or 38 or 39 or 40 | 1027887 |  |
|  |  |  |  |
| 44 | 41 and 42 and 43 | 1177 |  |
|  |  |  |  |
| 45 | limit 44 to (English language and embase and yr="2010 -Current") | 527 |  |
|  |  |  |  |

Detailed database search and results from Web of Science core collection (Science citation index and Social Science citation index) = 469

| 4 | #1 AND #2 AND #3 and 2011 or 2012 or 2013 or 2014 or 2015 or 2016 or 2017 or 2018 or 2019 or 2020 or 2021 or 2022 (Publication Years) and English (Languages) | 469 |
| --- | --- | --- |
| 3 | (TI=(train* or educat* or competenc*or skill or course or qualification or faculty or aptitude or capacity or curricul*)) OR AB=(train* or educat* or competenc*or skill or course or qualification or faculty or aptitude or capacity or curricul*) | 3,497,401 |
| 2 | (TI=( “Digital health” or “digital public health” or “digital tech*” or “health informatics” or mhealth or ehealth or e-health or "artificial intelligence" or "machine learning" or "big data" or "social media" or digitalization or "digital transformation" or "medical informatics" or "biomedical informatics" )) OR AB=( “Digital health” or “digital public health” or “digital tech*” or “health informatics” or mhealth or ehealth or e-health or "artificial intelligence" or "machine learning" or "big data" or "social media" or digitalization or "digital transformation" or "medical informatics" or "biomedical informatics" ) | 291,100 |
| 1 | (TI=(("Public health" or epidemiolog* or surveillance or “health promotion” or “health protection” or “health prevention” or “health policy” or “community health” or “health evaluation” or “emergency preparedness”))) | 253763 |

Detailed database search and results from ERIC = 252

| S2 | ((("Public health" or epidemiolog* or surveillance or “health promotion” or “health protection” or “health prevention” or “health policy” or “community health” or “health evaluation” or “emergency preparedness”))) ) AND ( (( “Digital health” or “digital public health” or “digital tech*” or “health informatics” or mhealth or ehealth or e-health or ""artificial intelligence" or "machine learning"" or "big data"" or "social media"" or digitalization or "digital transformation" or "medical informatics" “medical informatics" or "biomedical informatics" )) OR AB=( “Digital health” or “digital public health” or “digital tech*” or “health informatics” or mhealth or ehealth or e-health or "artificial intelligence" or "machine learning"" or "big data" or "social media"" or digitalization or "digital transformation"" or "medical informatics" or "biomedical informatics" )AND (train* or educat* or competenc*or skill or course or qualification or faculty or aptitude or capacity or curricul*)) OR AB=(train* or educat* or competenc*or skill or course or qualification or faculty or aptitude or capacity or curricul*) | Limiters - Published Date: 20100101-20221231; Language: English | 252 |
| --- | --- | --- | --- |
| S1 | ((("Public health" or epidemiolog* or surveillance or “health promotion” or “health protection” or “health prevention” or “health policy” or “community health” or “health evaluation” or “emergency preparedness”))) ) AND ( (( “Digital health” or “digital public health” or “digital tech*” or “health informatics” or mhealth or ehealth or e-health or ""artificial intelligence" or "machine learning"" or "big data"" or "social media"" or digitalization or "digital transformation" or "medical informatics" “medical informatics" or "biomedical informatics" )) OR AB=( “Digital health” or “digital public health” or “digital tech*” or “health informatics” or mhealth or ehealth or e-health or "artificial intelligence" or "machine learning"" or "big data" or "social media"" or digitalization or "digital transformation"" or "medical informatics" or "biomedical informatics" )AND (train* or educat* or competenc*or skill or course or qualification or faculty or aptitude or capacity or curricul*)) OR AB=(train* or educat* or competenc*or skill or course or qualification or faculty or aptitude or capacity or curricul*) |  | 268 |

**Supplementary search:**

Supplementary search was conducted in Google scholar, google, and other public health agency/public health training association websites including the Association of Schools and Programs of Public health (ASPPH), Public Health Agency of Canada (PHAC), World Federation of Public Health Associations (WFPHA), Association of Schools of Public Health (ASPHER) and World Health Organization and its regional office websites and United Nations agencies.

Preprints were identified from Europe PMC and OVID Embase indexing preprints.

For supplementary search, keywords such as digital health curriculum, digital public health training, digital health training, digital health integration, digital health competencies, digital health, e-health, public health training competencies, integrating digital technologies were used.

**Results from supplementary search including web search.**

| **Search conducted in** | **Results retrieved** | **Results retained** |
| --- | --- | --- |
| Google Scholar | 100 | 59 |
| Google | 100 | 20 |
| ASPPH | 17 | 3 |
| PHAC | 81 | 4 |
| WFPHA | 3 | 1 |
| ASPHER | 14 | 1 |
| WHO | 24 | 2 |
| CEPH | 2 | 0 |
| CDC (USA) First 10 pages searched | 200 | 5 |
| UN | 100 | 0 |
| Embase preprints | 7 | 7 |
| Europe PMC preprints | 28 | 28 |
| Total | 274 | 130 |
