# Supplementary material for: Digital Competencies and Training Approaches to Enhance the Capacity of Practitioners to Support the Digital Transformation of Public Health: Rapid Review of Current Recommendations
Source: JMIR Public Health Surveill. 2024 Sep 9;10:e52798. doi: 10.2196/52798 (PMC11403915; doi:10.2196/52798)
Supplement: Multimedia Appendix 3 [file publichealth-v10-e52798-s003.docx]

**APPENDIX – MMAT Assessment of studies**

| **MMAT Assessment** | | | | |
| --- | --- | --- | --- | --- |
| **Study ID** | **Are there clear research questions?** | **Do the collected data allow to address the research questions?** |  |  |
| Adewale 2022 | Yes | Yes | Quantitative - descriptive |  |
|  |  |  | Is the sampling strategy relevant to address the research question? | Yes |
|  |  |  | Is the sample representative of the target population?  supporting text | Yes |
|  |  |  | Are the measurements appropriate? | Yes |
|  |  |  | Is the risk of nonresponse bias low? | No |
|  |  |  | Is the statistical analysis appropriate to answer the research question? | Yes |
| Hsu 2012 | Yes | Yes | Quantitative - descriptive |  |
|  |  |  | Is the sampling strategy relevant to address the research question? | Yes |
|  |  |  | Is the sample representative of the target population?  supporting text | Yes |
|  |  |  | Are the measurements appropriate? | Yes |
|  |  |  | Is the risk of nonresponse bias low? | Yes |
|  |  |  | Is the statistical analysis appropriate to answer the research question? | Yes |
| Brownson 2015 | Yes | yes | Qualitative - KI interviews |  |
|  |  |  | Qualitative; Is the qualitative approach appropriate to answer the research question?  supporting text | Yes |
|  |  |  | Are the qualitative data collection methods adequate to address the research question? | Yes |
|  |  |  | Are the findings adequately derived from the data? | Can't tell |
|  |  |  | Qualitative; Is the interpretation of results sufficiently substantiated by data? supporting text | Yes |
|  |  |  | Is there coherence between qualitative data sources, collection, analysis and interpretation? | Can't tell |
| Kampov-Polevoi 2011 | Yes | Yes | Qualitative |  |
|  |  |  | Qualitative; Is the qualitative approach appropriate to answer the research question?  supporting text | Yes |
|  |  |  | Are the qualitative data collection methods adequate to address the research question? | Yes |
|  |  |  | Are the findings adequately derived from the data? | Can't tell |
|  |  |  | Qualitative; Is the interpretation of results sufficiently substantiated by data? supporting text | Yes |
|  |  |  | Is there coherence between qualitative data sources, collection, analysis and interpretation? | Can't tell |
| Beyene 2021 | Can't tell | Can't tell | N/A - Commentary/Perspective | |
| Brownson 2017 | Can't tell | Can't tell | N/A - Commentary | |
| Erwin 2017 | Can't tell | Can't tell | N/A - Commentary | |
| Joshi 2012 | Yes | Yes | N/A - Scan of Environment | |
| Joshi 2021 | Can't tell | Can't tell | N/A - Commentary | |
| Stellefson 2020 | Can't tell | Can't tell | N/A - Commentary | |
| Wholley 2018 | Can't tell | Can't tell | N/A - Commentary | |
| Yu 2015 | Yes | Can't tell | Cross-sectional; brief research communication | |
