# Supplementary material for: Digital Competencies and Training Approaches to Enhance the Capacity of Practitioners to Support the Digital Transformation of Public Health: Rapid Review of Current Recommendations
Source: JMIR Public Health Surveill. 2024 Sep 9;10:e52798. doi: 10.2196/52798 (PMC11403915; doi:10.2196/52798)
Supplement: Multimedia Appendix 4 [file publichealth-v10-e52798-s004.docx]

**APPENDIX – Data Extraction form**

| **General information** |  |
| --- | --- |
| Study ID |  |
| Title |  |
| Citation: Lead author contact details |  |
| Article type |  |
| Country in which the study conducted |  |
| Year of publication |  |
| Summary notes |  |
| **Characteristics of included studies** |  |
| Aims/objectives of study |  |
| Setting |  |
| **Data collection** |  |
| What types of data are collected/managed/shared? |  |
| How is this data collected/managed/shared? |  |
| Study design |  |
| Theoretical framework |  |
| Participants |  |
| Intended audience/target professions |  |
| Intended audience/target population |  |
| **Key findings/recommendations** |  |
| Key descriptive statistics |  |
| Digital technologies discussed |  |
| Public health domains described in relation to digital technologies |  |
| Training competency recommendations related to digital technologies and specific public health disciplines identified |  |
| Digital competency recommendations related to digital technologies and specific public health disciplines identified |  |
| Disciplines identified as relevant for training in using digital technologies for public health |  |
| Approaches to training and competency development identified |  |
| Other related findings |  |
| Reported limitations |  |
| Additional training and competency gaps discussed |  |
| **Assessment and impacts** |  |
| Strengths and Limitations (as noted by authors) |  |
| MMAT Assessment Results |  |
